# PTEN inhibitor improves vascular remodeling and cardiac function after myocardial infarction through PI3k/Akt/VEGF signaling pathway

**DOI:** 10.1186/s10020-020-00241-8

**Published:** 2020-11-19

**Authors:** Qiuting Feng, Xing Li, Xian Qin, Cheng Yu, Yan Jin, Xiaojun Qian

**Affiliations:** 1 Department of Cardiovascular, the Affiliated Wuxi No.2 People’s Hospital of Nanjing Medical University, No.68, Zhongshan Road, Wuxi, 214002 Jiangsu China; 2 Department of Respiratory, the Affiliated Wuxi No.2 People’s Hospital of Nanjing Medical University, No.68, Zhongshan Road, Wuxi, 214002 Jiangsu China

**Keywords:** PTEN inhibitor, Angiogenesis, Cardiac function, PI3K, Akt, VEGF, Apoptosis

## Abstract

**Background:**

Myocardial infarction (MI) is the leading cause of death from cardiovascular disease (CVD). Currently, the efficacy for MI treatment remains unsatisfactory. Therefore, it is urgent to develop a novel therapeutic strategy.

**Methods:**

Left anterior descending arteries (LAD) of mice were ligated to induce MI. Another set of mice were intravenously injected with PTEN inhibitor BPV (1 mg/kg) 1 h after LAD ligation and continued to receive BPV injection daily for the following 6 days. Mice were performed echocardiography 14 days after surgery.

**Results:**

Mice in MI group displayed an increased expression of PTEN with impaired cardiac function, enhanced cardiomyocyte apoptosis and decreased angiogenesis. BPV treatment significantly improved cardiac function, with reduced cardiomyocyte apoptosis, promoted angiogenesis, and activated PI3K/Akt/vascular endothelial growth factor (VEGF) signaling pathway.

**Conclusion:**

PTEN inhibitor BPV could effectively prevent myocardial infarction in mice, highlighting its potential as a candidate therapeutic drug.

## Background

Acute myocardial infarction (AMI) occurs when blood flow dramatically decreases or suddenly stops, which causes acute and persistent ischemia and hypoxia of coronary arteries, leading to severe impairment of cardiac function (Boateng and Sanborn 2013). Myocardial infarction (MI) is the leading cause of death from cardiovascular disease (CVD), which is the number one cause of death worldwide (Saleh and Ambrose 2018). Many cardiomyocytes necrosis in the infarcted hearts are eventually replaced by fibrous tissue, resulting in the loss of cardiac function (Bahit et al. 2018). According to the report of World Health Organization (WHO), there are 32.4 million myocardial infarctions globally every year. In addition, patients who underwent previous MI are at the highest risk for further coronary and cerebral events (Lu et al. 2015). Similarly, survivors from MI are more susceptible of recurrent infarctions (Schumacher et al. 1998). In fact, MI is an imbalance between blood supply in coronary and myocardial demand (Reddy et al. 2015; Ibanez et al. 2018). Currently, the most effectively therapeutic approach for MI is still percutaneous coronary intervention (PCI) and most medications for MI, including aspirin, thrombolytics, vasodilators, anti-platelets, anti-coagulants have unsatisfied outcome with certain side effects (Spath et al. 2016; Hermanides et al. 2018).

Due to the potential role of angiogenesis to rescue ischemic myocardium at early stage after MI, proangiogenic therapy becomes a novel strategy for treating MI patients (Cochain et al. 2013; Robich et al. 2011; Losordo 1998). Angiogenesis is a multi-step dynamic process, including endothelial cell proliferation, migration and eventually angiogenesis. Animal studies and clinical trials showed that most common angiogenic growth factors such as fibroblast growth factor (FGF), and vascular endothelial growth factor (VEGF) (Zhou et al. 2005; Pearlman et al. 1995) could induce de novo formation of micro-vessels and ameliorate cardiac function in the infarcted hearts, and among all these factors, VEGF is the most important one to trigger angiogenesis.

Phosphatase and tensin homolog (PTEN) acts as a tumor suppressor gene. By virtue of its lipid phosphatase activity, PTEN dephosphorylates phosphatidylinositol (PIP3) to generate phosphatidylinositol 4,5-bisphosphate (PIP2) and negatively regulates PI3K/Akt signaling pathway (Lee et al. 2018). PTEN was proved to participate in the progression of MI. For example, long noncoding RNA MALATI regulated acute MI through miR-320-PTEN axis (Hu et al. 2018). Inhibition of PTEN improved cardiac dysfunction and remodeling of MI by miRNA-130a (Zheng et al. 2019). Moreover, previous evidence reported that PTEN also regulated angiogenesis via PI3K/Akt/VEGF signaling pathway (Ma et al. 2009). Additionally, PTEN-deficiency mice displayed an enhanced protection on ischemia-induced injury and a reduced inflammation and myocardial remodeling after MI, therefore our study aimed to explore the role of PTEN inhibitor in regulating myocardial microenvironment to protect cardiac function and ameliorate MI.

## Materials and methods

### Mouse procedure

8-week old male C57BL/6 mice were purchased from Animal Center in Nanjing University (Nanjing, China). All the animal care and experimental procedures were approved by the Animal Care and Use Committee of the Affiliated Wuxi No.2 People’s Hospital of Nanjing Medical University. After accommodation for 1 or 2 weeks, the left anterior descending arteries (LAD) of mice were ligated to induce myocardial infarction as previously described (Song et al. 2019; Liu et al. 2019). Briefly, after anesthetized, mice were intubated and ventilated by a rodent ventilator (Shanghai Alcott Biotech Co., Shanghai, China), then LAD was ligated by an 8.0 suture followed by the thoracotomy. Myocardial ischemia was confirmed by visual inspection and alternations in electrocardiography. The same procedure except for the LAD ligation was performed on the sham group. Mice were sacrificed 1 week after MI surgery, and hearts were collected for measuring the expression of PTEN.

BPV(HOpic) (bisperoxovanadium 5-hydroxipyridine-2-carboxylic acid), a selective inhibitor of PTEN, was bought from Sigma-Aldrich. For BPV treatment, mice were randomly divided into three groups. One is sham operated, the other two were LAD ligated. Mice were intravenously injected with BPV (1 mg/kg) (MI + BPV) or PBS (phosphate-buffered saline, MI + Vehicle) 1 h after LAD ligation, then mice were receiving the injection of BPV or PBS daily for the following 6 days.

Mice were anaesthetized by 2% isofluran to perform echocardiography 14 days after MI surgery as previously described (Liu et al. 2019). M-mode images of left ventricles (LV) were obtained from Nillar pressure–volume system (MPVS-400) to analyze left ventricular ejection fraction (LVEF), left ventricular end diastolic pressure (LVEDP) and hemodynamic parameter + dp/dt_max_. Data of pressure–volume loop was recorded at steady state during injection of hypertonic saline, and analyzed for the calibration of parallel conductance volume, which can be used to calculate LV volume of each mice as correction. Then mice were sacrificed, and hearts were collected to evaluate fibrosis by Masson’s trichrome staining as previously described (Yan et al. 2017). The areas, which were stained blue, were considered as scar tissue, while dark-red colored areas as viable myocardium. Area measurement was analyzed by Image J software to evaluate the scar area of the total area for each group.

### Immunohistochemical staining

The hearts from all groups of mice were dissected, fixed in 4% formalin, and embedded in paraffin. Transverse sections of peri-infarct area of the hearts were stained with rabbit-anti-PTEN, rabbit-anti-α-smooth muscle actin, rabbit-anti-CD31 respectively after deparaffinized and rehydrated overnight at 4 °C following by the incubation of anti-rabbit HRP antibody at room temperature for 1 h, and detected with 3,3′-diaminobenzidine. Images were taken using Nikon microscope and calculated as published before (Song et al. 2019). All the antibodies were purchased from Abcam, UK.

### Immunofluorescent staining

Paraffin-embed sections of peri-infarct area of the hearts from all groups of mice were deparaffinized and dehydrated, and incubated with anti-CD31 primary antibody overnight at 4 °C after blocking with 3% sheep serum. After washing with PBS, sections were incubated with Alexa-Fluor-coupled secondary antibody at room temperature for 60 min following with DAPI staining. All the antibodies were purchased from Invitrogen. Images were captured and analyzed using Leica microscope (Yu et al. 2017).

### TUNEL staining

Terminal deoxynucleotidyl transferase dUTP nick-end labeling (TUNEL) assay was used to analyze apoptotic cells using ApopTag Plus Fluorescem in Situ Apoptosis Detection Kit (Millipore) as previously described (Liu et al. 2019). At the same time, cardiac sections were also stained with α-actinin for 1 h at room temperature (Cell Signaling Technology, USA) for cardiomyocytes following with DAPI staining for the nuclei, then slides were detected under the Leica fluorescence microscope with four random fields of view in infarcted areas of each slide, and quantified at 200 magnification.

### Western blot

Protein were extracted from hearts of mice using Protein Extraction Kit (Beyotime Biotechnology, Beijing, China). After measuring the protein concentration by a BCA assay Kit (Thermo Scientific), the same amounts of protein were loaded, run in the SAS-PAGE gels, and transferred to PVDF membrane. Membranes were incubated with corresponding primary antibodies respectively at 4 °C overnight after blocking in 5% milk, followed by 1 h incubation of secondary antibody at room temperature. ECL reagents were applied to detect the bands, which were visualized by Bio-rad ChemiDoc. Images were analyzed by Image J software and normalized to GAPDH (Liu et al. 2012).

### Statistical analysis

Data were analyzed by statistical product and service solutions (SPSS 16.0, SPSS Inc,. Chicago, IL, USA) and expressed as means ± SEM. Student *t*-test, or one-way ANOVA analysis followed by a Tukey’s post hoc test was used to compare the differences between groups.

## Results

### The expression of PTEN was increased in the infarcted hearts of mice after MI

**Fig. 1:**
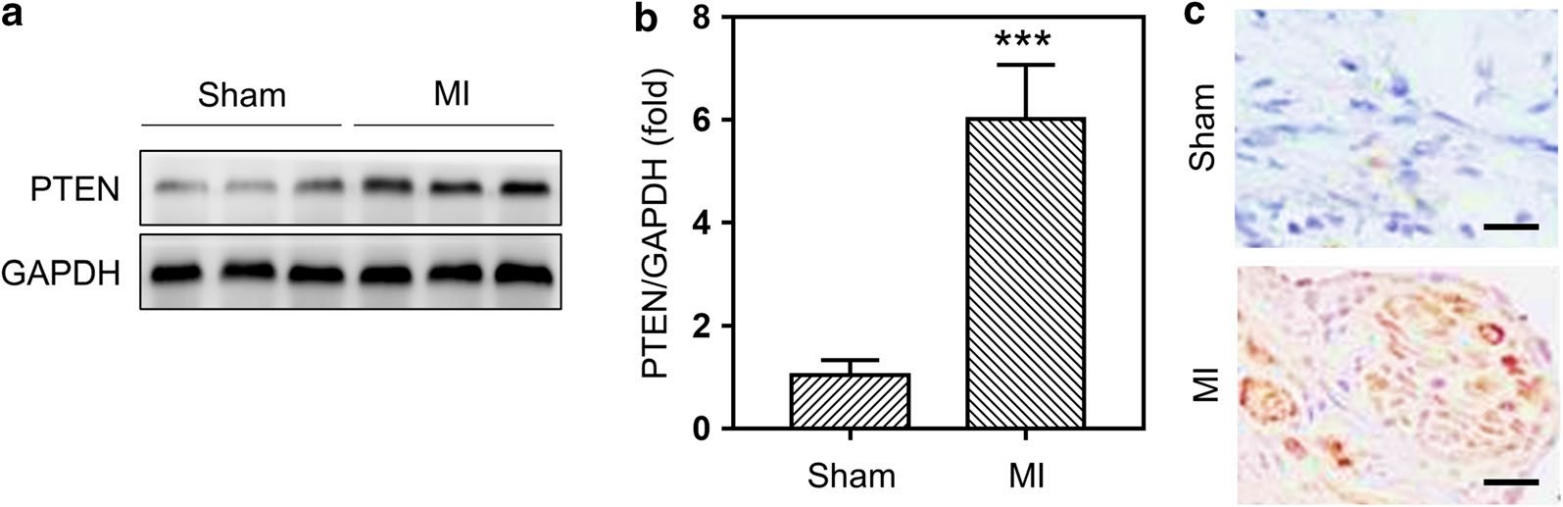
The expression of PTEN was increased in the infarcted hearts of mice after MI. **a**, **b** Western blot detected the protein levels of PTEN in the hearts of mice 7 days after MI surgery. Sham-operated mice were set as control. Three bands were representative of three different mice per group. Data was represented as means ± SEM. ***p < 0.001, n = 8, versus Sham mice. **c** Representative immunohistochemical images of PTEN in sham-operated wild type and MI mice

**Fig. 2:**
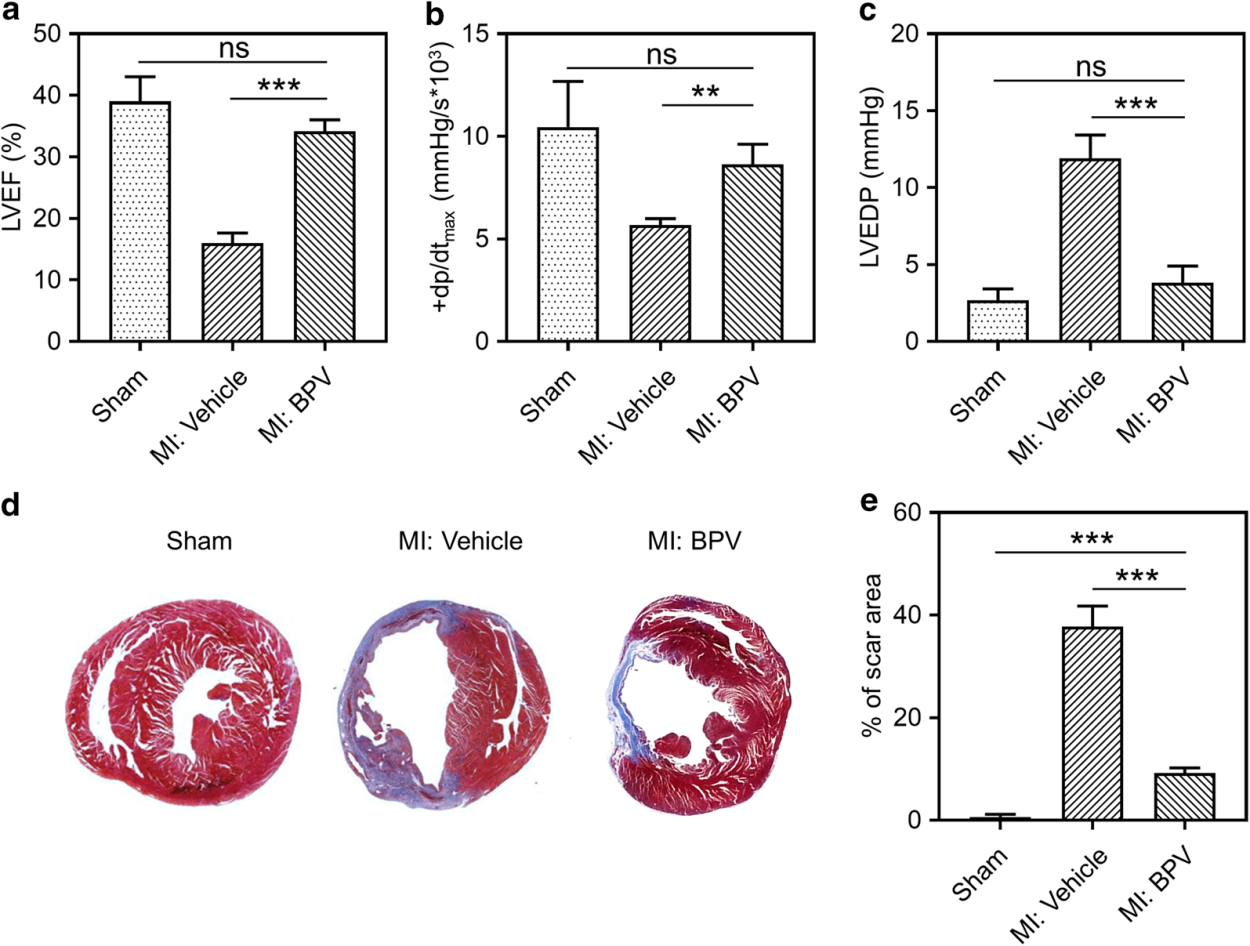
PTEN inhibitor improved cardiac functional in MI mice. Mice were injected intravenously with BVP (a selective inhibitor of PTEN, 1.0 mg/kg) 1 h after LAD ligation. MI mice were then administrated daily in the following 6 days. The effects of PTEN inhibitor on cardiac were examined on day 14 by Echocardiographic analysis in different groups. **a** Left ventricular ejection fraction (LVEF). Hemodynamic parameter + dp/dtmax (**b**) and LV end diastolic pressure (LVEDP) (**c**). n = 8 for each group. **d** Representative Masson’s trichrome-stained myocardial sections in the treated MI hearts. Blue, scar tissue; dark-red, viable myocardium. **e** Quantitation of infarcted size in the three indicated experimental groups. Data were represented as means ± SEM. **p < 0.01, ***p < 0.001, *ns* no significant difference, n = 8 for each group

To evaluate the role of PTEN in MI, we first measured the expressions of PTEN in the hearts of post-MI mice. Compared to sham-operated mice, Western blot data showed that protein levels of PTEN in the infarcted hearts of post-MI mice were significantly increased both 1 week (Fig. 1a, b) and 2 weeks after surgery (Additional file 1: Figure S1). In addition, immunohistochemical staining also indicated that the expression of PTEN was elevated in the hearts of MI mice compared to sham group (Fig. 1c). These data suggested that MI operation induced the expressions of PTEN in the infarcted hearts of mice.

### PTEN inhibitor improved cardiac function in MI mice

Next, to further evaluate the role of PTEN in MI, we applied a selective PTEN inhibitor BPV in MI mice. Compared to sham-operated mice, MI mice displayed an impaired cardiac function, as judged of decreased LVEF, + dp/dt_max_ and LV pressure–volume loops (Additional file 1: Figure S2) as well as increased LVEDP (Fig. 2a–c), which all confirmed the success of MI surgery. However, after receiving BPV (1 mg/kg) daily for 1 week, cardiac function of MI mice was significantly improved as judged of remarkably increased LVEF, + dp/dt_max_ and LV pressure–volume loops as well as reduced LVEDP. Moreover, Masson’s trichrome staining showed that the infarcted areas were elevated in MI mice compared to sham group, which were diminished by BPV treatment (Fig. 2d, e). All these data suggested that LAD ligation impaired cardiac function and caused MI in mice, which could be effectively restored by BPV treatment.

### PTEN inhibitor reduced cardiomyocyte apoptosis in MI mice

**Fig. 3:**
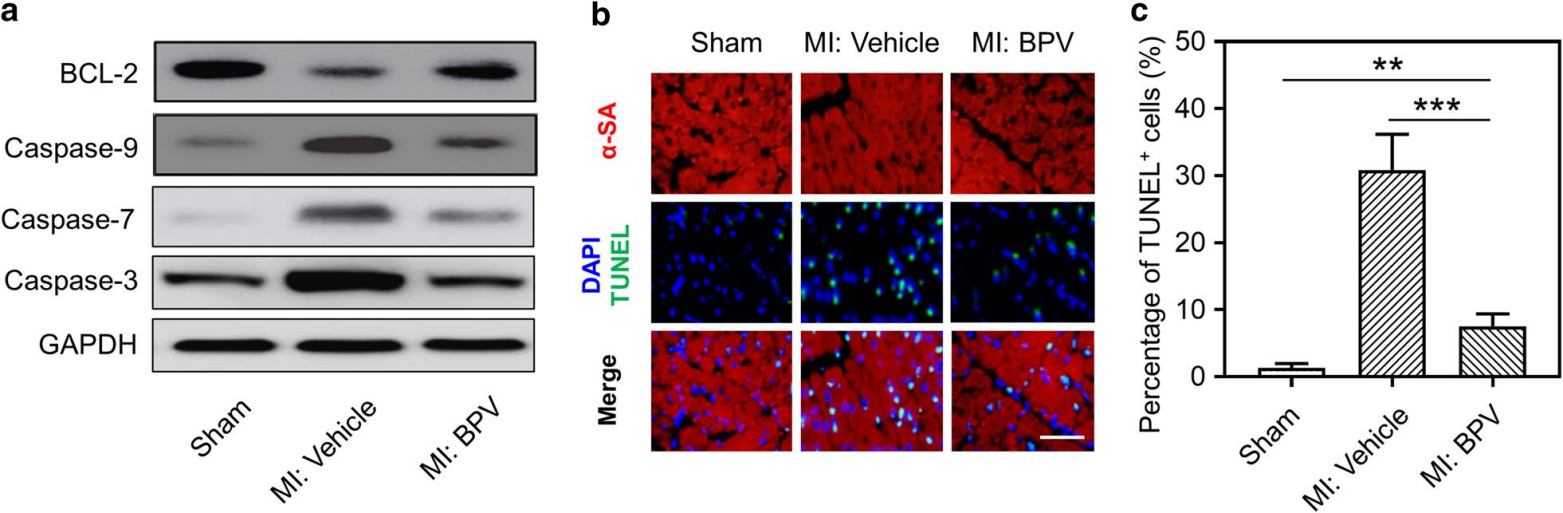
PTEN inhibitor reduced cardiomyocytic apoptosis in MI mice. **a** Western blot was used to detect the protein levels of Bcl2, Caspase 9, Caspase 7 and Caspase 3 the peri-infarct area of the heart 14 days after MI. **b**, **c** Representative TUNEL and α-actinin staining of cardiomyocytes in cardiac sections from different groups. Green color in TUNEL staining was apoptotic cells; red color represented α-actinin, which is the marker of cardiomyocytes; DAPI stained blue color was the nucleus. Scale bar = 50 μm. n = 8. Data were represented as means ± SEM. **p < 0.01, ***p < 0.001, *ns* no significant difference

Cardiomyocytes are susceptible to apoptosis during MI, and cardiomyocyte apoptosis is implicated to be involved in the pathological process of MI-induced tissue damage (Krijnen et al. 2002). In light of BPV improving cardiac function, we speculated whether PTEN inhibitor BPV could ameliorate MI-induced cardiomyocyte apoptosis. We first measured the inhibitory effect of BPV on PTEN expression. It showed that PTEN expression were significantly increased after MI operation, which was further remarkably inhibited by BPV treatment 7 days and 14 days post MI (Additional file 1: Figure S3). Next, Western blot data showed that the expression of Cysteine-aspartic proteases-3 (Caspase-3), Caspase-7, Caspase-9 was elevated, while B cell lymphoma 2 (BCL-2) expression was decreased, in the cardiomyocytes of MI mice, compared to those in sham-operated mice. Their expression could be partially and significantly rescued by BPV treatment (Fig. 3a). Moreover, immunofluorescent images also indicated that cardiomyocyte apoptosis, which was stained both TUNEL and α-actinin (cardiomyocyte marker), was increased in MI mice, and this increased apoptosis was remarkably decreased after BPV injection (Fig. 3b, c). It should be noted that few apoptotic cardiomyocytes were observed in BPV-treated sham mice (Additional file 1: Figure S4), which suggested that BPV treatment had no obvious effect on cardiomyocyte apoptosis. Taken together, BPV could effectively inhibit cardiomyocyte apoptosis in MI mice.

### PTEN inhibitor promoted angiogenesis in MI mice

**Fig. 4:**
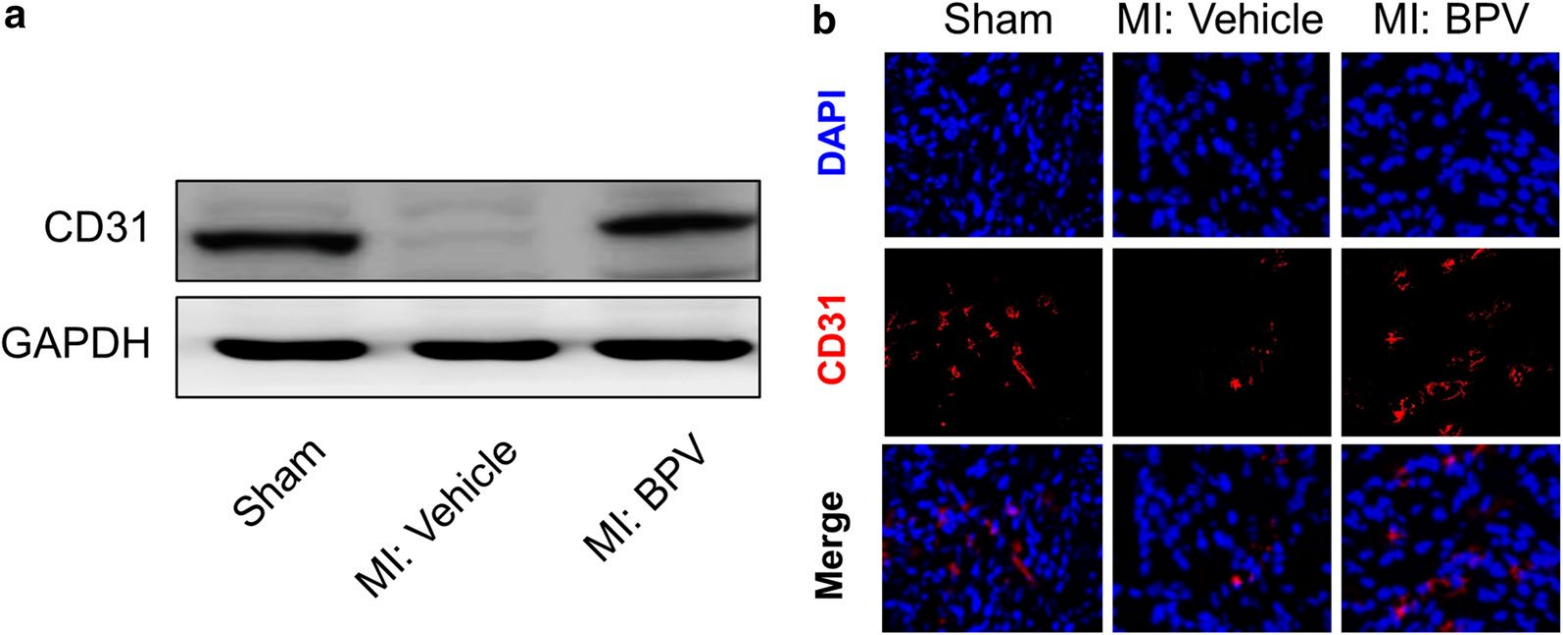
PTEN inhibitor promoted angiogenesis in MI mice. The expressions of CD31 in the hearts of the sham and BPV/vehicle-treated MI mice 14 days after MI, were examined by Western blot (**a**) and immunofluorescence staining (**b**). Immunofluorescent staining with anti-CD31 antibody showed the capillary densities in the infarcted heart 14 days after MI

The adhesive interactions among endothelial cells and the adhesion receptors in the process of angiogenesis are of fundamental importance to MI (DeLisser et al. 1997; Kobayashi et al. 2017). We therefore explored the effect of PTEN inhibitor BPV in the angiogenesis after MI. Here, we assessed the expression of cluster of differentiation 31 (CD31), also known as platelet endothelial cell adhesion molecule (PECAM-1), which makes up a large portion of endothelial cell intercellular functions. Both Western blot and immunofluorescent staining showed that the expressions of CD31 were remarkably decreased in MI mice compared to sham-operated mice, and this decline was significantly restored after BPV treatment 14 days after MI surgery (Fig. 4a, b). These results indicated that MI impaired capillary densities, and this impairment of capillary densities in the infarcted hearts could be ameliorated by BPV.

### PTEN inhibitor activated PI3K/Akt/VEGF signaling pathway in MI mice

**Fig. 5:**
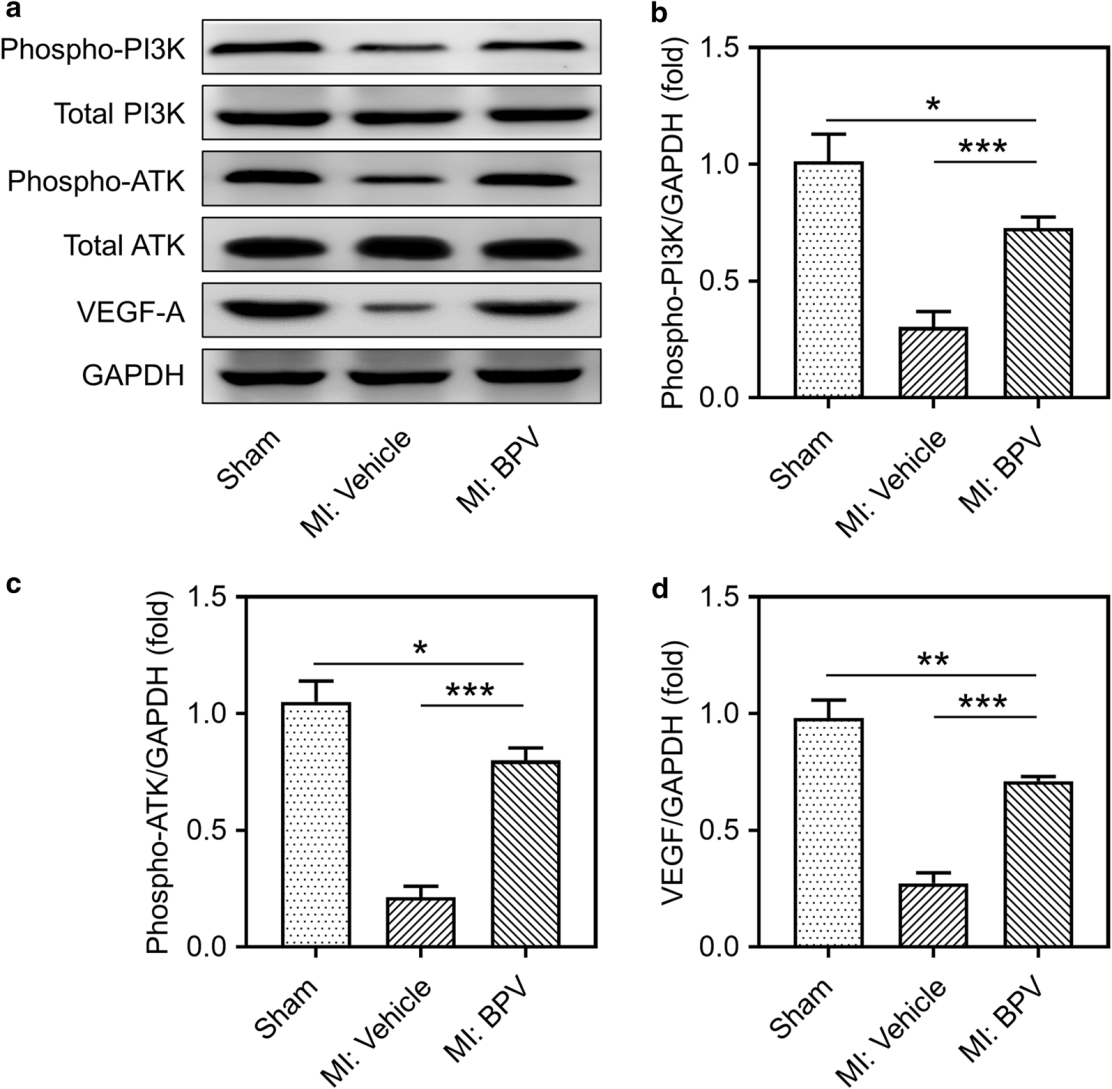
PTEN inhibitor activated PI3K/Akt/VEGF signaling pathway in MI mice. PTEN inhibitor activated PI3K and Akt signaling pathway (**a**–**c**) and induced the expression of VEGF-A in the hearts 14 days after MI (**a**, **d**). Data were represented means ± SEM. *p < 0.05, **p < 0.01, ***p < 0.001, *ns* no significant difference, n = 8

Previous studies demonstrated that PTEN regulated angiogenesis via PI3K/Akt/VEGF signaling pathway (Ma et al. 2009), therefore we speculated that PTEN inhibitor BPV also regulated angiogenesis in MI mice via PI3K/Akt/VEGF signaling pathway. Here, we found that the expressions of phosphorylated-PI3K, phosphorylated-Akt and vascular endothelial growth factor A (VEGF-A) were all reduced in the infarcted hearts of MI mice compared to sham group, and all of these decreased expressions were restored after BPV treatment in MI mice (Fig. 5a–d). These data suggested that PTEN inhibitor BPV stimulated PI3K/Akt/VEGF signaling pathway, which was deactivated in the infarcted hearts of MI mice.

## Discussion

Myocardial infarction happens when one of the coronary arteries in the heart is suddenly blocked or has extremely slow blood flow. Sudden blockage in a coronary artery is due to the formation of thrombus, which usually forms inside a coronary artery that already narrowed by atherosclerosis. And slow blood flow in a coronary artery occurs when demand for oxygen is greater than the supply, which is commonly called ischemia. Either blockage or slow blood flow in any coronary artery could cause malfunction and pain in the artery-corresponded parts of heart. Ischemia-caused tissue damage, myocardial necrosis and cardiomyocyte loss depend on several factors, including the size of the initial infarct, duration of ischemia and efficiency of reperfusion. MI is one of the leading causes of death and disability all over the world. Despite a variety of therapeutic strategies in clinical to treat MI patients, including reperfusion therapy, percutaneous transluminal coronary angioplasty (PTCA), anti-platelet drugs, such as aspirin and clopidogrel, and clot-dissolving drugs called thrombolytic agents, such as tissue plasminogen activator (tPA), the outcome remains unsatisfactory in current cardiovascular medicine. Because some patients remain ineligible for these therapies, their ischemic hearts with microvascular rarefaction and malfunction could prevent effective reperfusion of the entire myocardium. Therefore, prevention of recurrent infarction, recovery of cardiac function and blockage of the transition to heart failure in patients with MI is still a big challenge, urgent demand for developing novel therapeutic alternatives to treat MI patients (Shah and Mann 2011).

In the past half a century, proangiogenic therapy, which promotes angiogenesis in the infarcted hearts became a promising therapeutic strategy, because it enhances reperfusion in the ischemic hearts to improve cardiac function. Angiogenesis is a multi-step process of de novo formation of micro-vessels from pre-existing capillaries. Resting endothelial cells transited into an active form after receiving angiogenic signals, including hypoxia, angiogenic growth factors or nitric oxide (NO). Angiogenesis facilitates in rescuing ischemic myocardium at early stages after MI, recovering cardiomyocyte growth, survival and contractile function, and promoting chronic left ventricular remodeling to prevent the transition to heart failure. Therefore, more and more attentions were paid on exploring the role of angiogenesis for treating patients with MI. However, until now, promising preclinical studies failed to meet the expectation in clinical trails (Losordo and Dimmeler 2004; Tongers et al. 2011), which further expands demand for the development of new proangiogenic drugs. Therefore, our current study aimed to evaluate a new proangiogenic drug in MI, which is PTEN inhibitor BPV.

PTEN is a highly conserved dual-specificity protein tyrosine phosphatase (PTP). As a tumor suppressor gene, PTEN is capable to inhibit the progressions of numerous tumors, from benign to the most malignant forms, in which angiogenesis was participated, namely angiogenic switch. Increasing evidence demonstrated that PTEN participated in the progression of myocardial infarction (Mahmoud et al. 2019; Wu et al. 2019), and patients with acute MI had increased levels of PTEN in the serum, which suggested that PTEN might be used as predict marker for MI. Moreover, varieties of candidates improved MI by regulating PTEN/PI3K/Akt pathway, such as miR-320 (Hu et al. 2018), miR-26a (Zhang and Cui 2018), miR-144-3p (Yuan et al. 2019) and Astragaloside IV (Cheng et al. 2019). Moreover, lots of publications implicated that PTEN inhibited tumor-induced angiogenesis through PI3K/Akt signaling pathway (Ma et al. 2009; Wen et al. 2001; Fang et al. 2007; Jiang and Liu 2008, 2009; Zhong et al. 2000). In addition, there were some studies demonstrated that PTEN regulated VEGF-mediated angiogenesis, which is a crucial angiogenic growth factor (Ma et al. 2009; Huang and Kontos 2002; Koul et al. 2002). Due to its inhibitory effect on angiogenesis, we speculated that PTEN inhibition could be feasible as a proangiogenic strategy.

Nowadays, accumulating evidences proved that PTEN inhibition-based therapies in human diseases were feasible (Pulido 2018). Besides PTEN-deficiency mice, several compounds were applied to inhibit PTEN. Different forms of BPV as well as VO-OHpic (hydroxyl(oxo)vanadium 3-hydroxypiridine-2-carboxylic acid) have been extensively used as PTEN specific inhibitors. Some animal studies proved AKT-dependent negative role of PTEN in nerve growth and regeneration (Danilov and Steward 2015; Du et al. 2015; Jin et al. 2015). Moreover, PTEN inhibitor BPV was reported to be beneficial for nerve regeneration and neurosurvival-related diseases in animal models. For example, BPV (HOpic) treatment reduced apoptosis of hippocampus neuronal cells in the mouse model of Alzheimer’s disease (AD). In addition, PTEN inhibitors were also implicated to promote wound healing, tissue maintenance, and caner-related diseases (Hu et al. 2010; Mihai et al. 2012; Augello et al. 2016). In line with PTEN inhibition on ischemia or reperfusion-caused cardiac damage, PTEN-deficiency mice displayed an enhanced protection on ischemia-induced injury and a reduced inflammation and myocardial remodeling after MI (Parajuli et al. 2012). This study gave us a hint that PTEN inhibitor might have the similarly protective effect on MI as deletion of PTEN in mice. Moreover, in parallel with increased protection to pathological hypertrophy in PTEN gene-deleted cardiomyocytes. Thus, we excluded that PTEN inhibitor might protect against MI in mice, but so far, there is no publication about that, so in our current study, we applied PTEN inhibitor BPV to treat MI mice, and our data confirmed our hypothesis.

In summary, our study for the first time explored the PTEN inhibitor BPV as a proangiogenic drug to protect against MI in mice, and this might give us a hint that PTEN inhibitor BPV might serve as a candidate therapeutic drug to treat MI patients in the future.

## Conclusion

Our study for the first time explored the PTEN inhibitor BPV as a proangiogenic drug to protect against MI in mice, and this might give us a hint that PTEN inhibitor BPV might serve as a candidate therapeutic drug to treat MI patients in the future.

## Supplementary information

**Additional file 1: Figure S1.** Western blot detected the protein levels of PTEN in the hearts of mice 14 days after MI surgery. Sham-operated mice were set as control. **Figure S2.** Representative LV pressure–volume loops acquired from Sham, MI-Vehicle and MI-BPV mice. **Figure S3.** The protein levels of PTEN 7 days and 14 days post first dose were examined, and the PTEN levels were much lower in the BVP-treated group, than the Vehicle-treated group. **Figure S4.** Representative TUNEL staining on cardiac myocytes in cardiac sections from BPV-treated Sham mice. Green color was TUNEL staining representing apoptotic cells, blue color was the cell nucleus stained by DAPI. Scale bar = 50 μm.

## Data Availability

The raw data supporting the conclusions of this manuscript will be made available by the authors, without undue reservation, to any qualified researcher.
