# Supplementary material for: PTEN inhibitor improves vascular remodeling and cardiac function after myocardial infarction through PI3k/Akt/VEGF signaling pathway
Source: Mol Med. 2020 Nov 19;26:111. doi: 10.1186/s10020-020-00241-8 (PMC7678076; doi:10.1186/s10020-020-00241-8)
Supplement: Supplementary file 1 — Additional file 1: Figure S1. Western blot detected the protein levels of PTEN in the hearts of mice 14 days after MI surgery. Sham-operated mice were set as control. Figure S2. Representative LV pressure–volume loops acquired from Sham, MI-Vehicle and MI-BPV mice. Figure S3. The protein levels of PTEN 7 days and 14 days post first dose were examined, and the PTEN levels were much lower in the BVP-treated group, than the Vehicle-treated group. Figure S4. Representative TUNEL staining on cardiac myocytes in cardiac sections from BPV-treated Sham mice. Green color was TUNEL staining representing apoptotic cells, blue color was the cell nucleus stained by DAPI. Scale bar = 50 μm. [file 10020_2020_241_MOESM1_ESM.docx]

**Additional information**

**Figure S1.** Western Blot detected the protein levels of PTEN in the hearts of mice 14 days after MI surgery. Sham-operated mice were set as control.


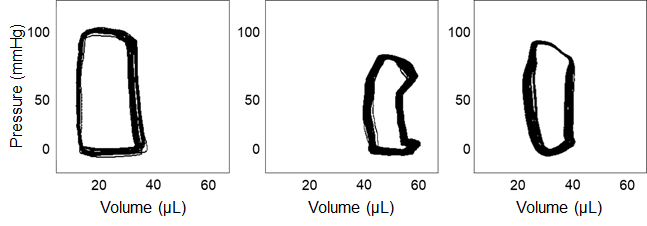


**Figure S2.** Representative LV pressure-volume loops acquired from Sham, MI-Vehicle and MI-BPV mice.

**Figure S3.** The protein levels of PTEN 7 days and 14 days post first dose were examined, and the PTEN levels were much lower in the BVP-treated group, than the Vehicle-treated group.

**Figure S4.** Representative TUNEL staining on cardiac myocytes in cardiac sections from BPV-treated Sham mice. Green color was TUNEL staining representing apoptotic cells, blue color was the cell nucleus stained by DAPI. Scale bar = 50 μm.
